# Femoral artery variation was found during V-A ECMO catheterization

**DOI:** 10.1186/s13019-022-01982-9

**Published:** 2022-08-31

**Authors:** Liwen Du, Leilei Zhu, Yongwei Shi, Peng Liu, Kai Xun

**Affiliations:** 1 Emergency Department, Hwa Mei Hospital, University of Chinese Academy of Sciences, Ningbo, 315010 China; 2 Ningbo Institute of Life and Health Industry, University of Chinese Academy of Sciences, Ningbo, 315010 China

**Keywords:** V-A ECMO, Arterial cannulation, Deep femoral artery, Superficial femoral artery, Distal limb perfusion

## Abstract

**Background:**

High bifurcation of the deep femoral artery (DFA) is rare in clinical practice, and patients with this variation are less likely to receive venoarterial extracorporeal membrane oxygenation (V-A ECMO) treatment. Therefore, the method by which V-A ECMO is introduced in patients with vascular variation is very important.

**Case presentation:**

A 52-year-old male patient had ST elevation myocardial infarction due to coronary heart disease. Angiography showed tripartite coronary artery lesions, and coronary artery stenting supported by V-A ECMO was needed. Vascular evaluation before ECMO catheterization revealed high bifurcation of the bilateral DFA located at the inguinal ligament. After discussion, the perfusion cannula was placed in the left superficial femoral artery (SFA) towards the heart, and the distal perfusion catheter (DPC) was placed in the left SFA towards the distal end. Nevertheless, after the patient's heart recovered, necrosis of the toe of the left lower limb still occurred.

**Conclusion:**

Common femoral artery assessment must be performed before V-A ECMO for patients with high bifurcation of the DFA. Incision catheterization and DPC placement are recommended. After decannulation, arterial repair under direct visualisation is recommended, and rigorous distal vascular assessment and management are needed.

## Background

The deep femoral artery (DFA) is the largest branch of the common femoral artery (CFA); it supplies blood to the medial circumflex femoral artery (MCFA), lateral circumflex femoral artery (LCFA), thigh muscles, hip joint, and femur. Vascular variations at this location need to be considered during interventional and surgical procedures [1]. Venoarterial extracorporeal membrane oxygenation (V-A ECMO) is a temporary circulatory support strategy for the treatment of cardiac arrest or cardiopulmonary failure, and its indications are expanding [2]. Percutaneous catheterization does not require thoracotomy but carries a risk of vascular complications during rapid establishment. A patient with peripheral cannulation is described below, and his CFA variation led to nonstandard catheterization. Ischemia and necrosis of the toe at the ipsilateral end of the arterial cannula appeared after decannulation. The article was written in accordance with the CARE reporting checklist.

## Case presentation

A 52-year-old male patient had been suffering from hypertension and diabetes for more than 10 years. His blood pressure and glucose were not well controlled under normal circumstances, and the second toe of the right foot was removed because of diabetes. He went to a subordinate hospital because of "chest tightness for 3 days and aggravation for 1 h". His troponin level was 1.78 ng/ml, echocardiography indicated an ejection fraction (EF) of 35%, and ECG showed extensive elevation of the ST segment on the anterior wall lead. The diagnosis was considered ST-segment elevation myocardial infarction (STEMI), cardiogenic shock (CGS), and Killip IV. The local hospital immediately performed coronary angiography, which indicated tripartite coronary artery lesions. Due to the high vasoactive inotrope score (VIS), the doctor thought that there was a high risk of malignant arrhythmia during vascular opening, so the patient was transferred to our hospital.

**Fig. 1:**
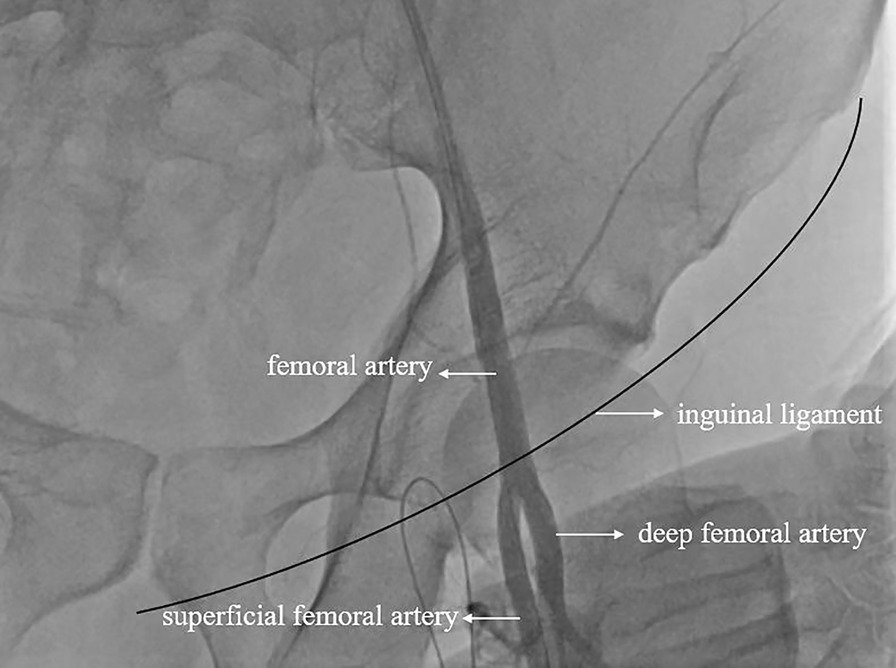
High bifurcation of bilateral deep femoral artery (DFA) angiographic evidence

The diagnosis of STEMI was clear. After consensus was reached between the cardiology department and the emergency ECMO team, V-A ECMO interventional support was planned. Before ECMO catheterization, ultrasound was used for vascular evaluation, and the femoral vein (FV) showed no obvious variation. However, no DFA bifurcation was found under the inguinal ligament, so the team decided to use a skin incision for catheterization. Due to vascular variation, isolating the FA without opening the inguinal ligament was impossible. Given the presence of CGS at that time, the patient needed emergency percutaneous coronary intervention (PCI), so the perfusion cannula was first placed into the superficial femoral artery (SFA) after weighing the advantages and disadvantages. The Seldinger method was used to place the guide wire directly into the SFA to obtain angiographic evidence, as shown in Fig. 1. Then, in a stepwise manner, the cannula (Maquet®, 23 cm, 15F) was placed into the SFA at a depth of 15 cm to expand the channel. The drainage cannula (Maquet®, 39 cm, 21 F) was routinely placed in the FV by traditional percutaneous catheter placement. After the pipeline was established and positioned via ultrasonic guidance, V-A ECMO was administered at a pump speed of 3000 RPM, flow rate of 2.8 L/min and target mean arterial pressure (MAP) > 79 mmHg.

Coronary angiography was performed with ECMO support. Angiography showed long segment stenosis in the middle segment of the left main trunk and 95% stenosis at the heaviest point. The circumflex branch was completely occluded below the proximal end. Distal phantom development was provided by the lateral and right crown branches. The above vessels were dilated and shaped by a balloon, and then a stent was implanted. Transient ventricular tachycardia and ventricular fibrillation occurred several times during the operation. When the team briefly adjusted the ECMO speed, the heart rate recovered, and MAP remained stable. After coronary stenting, the team decided to continue in vitro circulation support to avoid cardiogenic shock after myocardial reperfusion due to frequent arrhythmias during surgery and a high VIS. As the CFA variation was clear, the patient was returned to the ICU with V-A ECMO after the distal perfusion catheter (DPC) was inserted in the digital subtraction angiography (DSA) room.

On the first day after DSA, ECMO ran at a speed of 2500 RPM and a flow rate of 2.0 L/min. When the anticoagulant target was reached, the pressure of the DPC increased, and the fluctuation in the left dorsal foot artery was significantly weakened. The patient was at high risk of thrombosis in the distal left lower extremity. Given the patient's stable haemodynamic status, after the withdrawal test was passed, it was decided through multidisciplinary consultation that left CFA repair and SFA thrombectomy would be performed simultaneously with the removal of V-A ECMO. Postoperatively, the left CFA, popliteal artery and dorsalis pedis artery were all pulsing. After the patient returned to the ward, heparin was continued for 12 h, followed by low molecular weight heparin sodium 4250 IU q12 h IH, aspirin 100 mg qd bs, and clopidogrel 75 mg qd bs antiplatelet therapy.

**Fig. 2:**
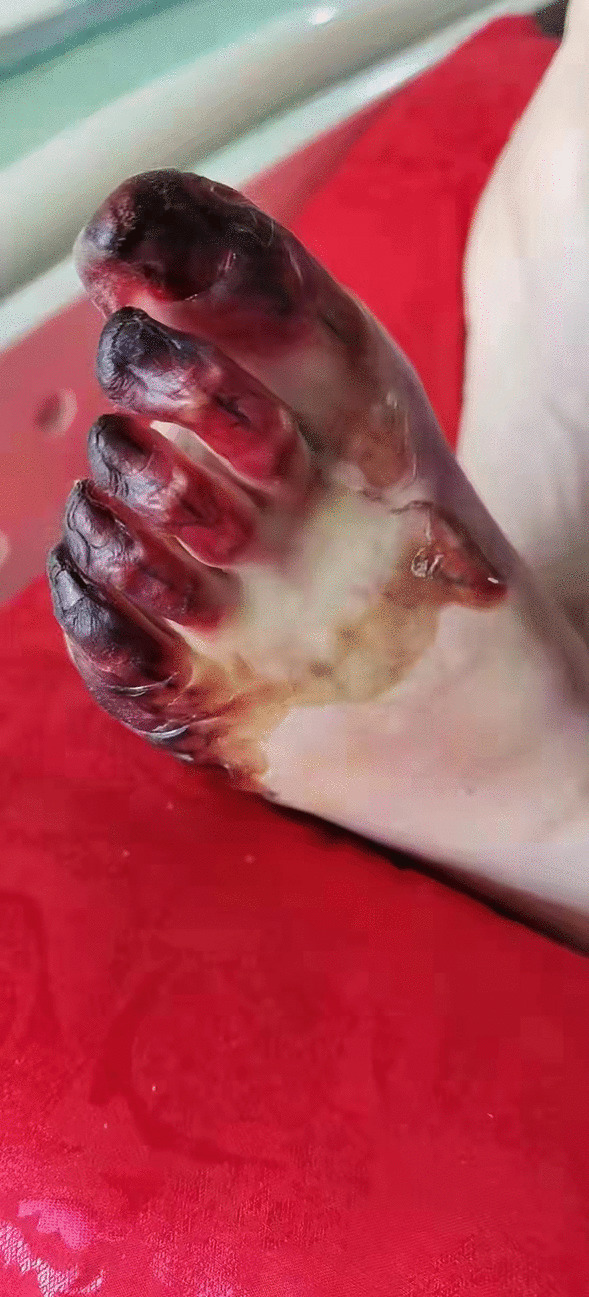
Tissue blackening gangrene

**Fig. 3:**
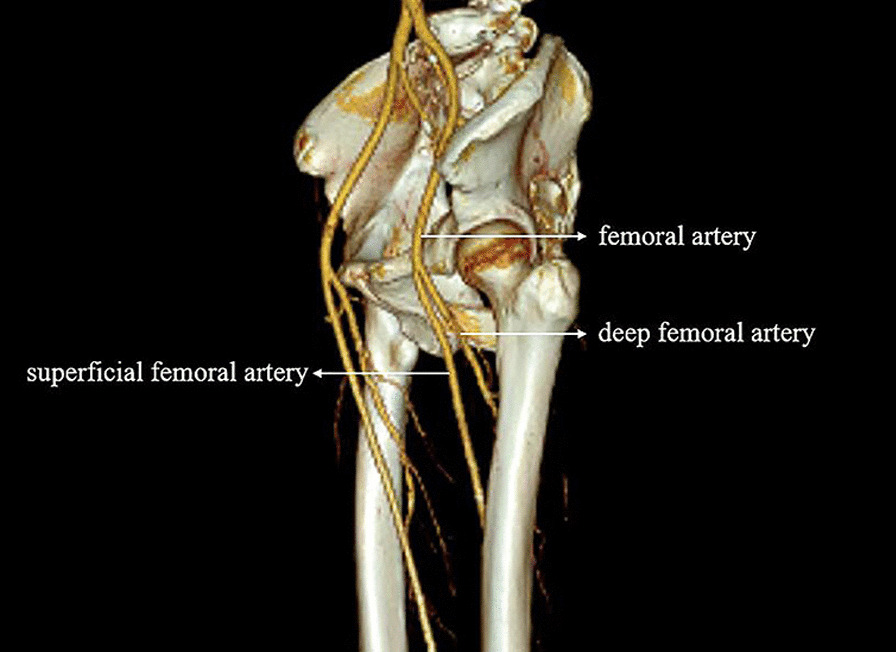
High bifurcation of the DFA was observed in both lower limbs

On the third day after the removal of ECMO, the patient was released from mechanical ventilation, and cardiac function recovered significantly. Tension blisters gradually appeared on the patient's left lower limb, which later progressed to gangrene with skin stripping and tissue blackening, as shown in Fig. 2. Computed tomography angiography (CTA) examination of the blood vessels in both lower extremities was performed. The report suggested significant atherosclerotic changes in the arteries in both lower limbs with varying degrees of stenosis. High bifurcation of the DFA was observed in both lower limbs, and CTA reconstruction is shown in Fig. 3. After consultation with orthopaedic and vascular surgery, it was decided to amputate the entire toe on the left foot considering the patient's past medical history. After comprehensive nursing care, the left foot healed well, and the patient was discharged from the hospital after his general condition was stable. At the time of discharge, the patient's cardiac function had recovered to New York Heart Association (NYHA) grade I, and the Karnofsky performance score (KPS) was 80. The presentation of all case data was authorized by the patient and his family.

## Discussion

The DFA originated posterolateral of the CFA 2.5–5.5 cm below the inguinal ligament (76%), passing deep between the adductor longus and pectineus muscles. A meta-analysis by Tomaszewski et al. reported that the DFA most commonly originates in the proximal third of the thigh (47.6%). The mean gathering distance from the mid-inguinal point (MIP) was 41.2 mm [3].

The prognosis of ECMO patients is affected not only by the severity of the patient's condition, the type of disease and other organ functions but also by the related complications. Common complications of V-A ECMO include thrombosis (1–22%), haemorrhage (5–79%), infection (17–49%), and limb ischaemia (13–25%) [4]. The cannula required for V-A ECMO is usually placed peripherally using the Seldinger method; the venous cannula is usually placed in the FV for drainage, and the arterial cannula is usually placed in the CFA (not SFA) for perfusion. This method is much more convenient and faster than central catheterization. Although a cannula that is two-thirds the diameter of the common femoral artery is used, adult catheterization using the CFA for ECMO support is still often accompanied by SFA obstruction leading to limb ischaemia.

Our ECMO centre experience, combined with existing research, shows that a small FA diameter, high VIS, and history of high risk of lower extremity vascular necrosis (such as diabetic foot, peripheral arterial occlusive disease, high blood coagulation, etc.) are all high-risk factors for distal limb ischaemic necrosis [5]. Prophylactic placement of a DPC would be considered in all of these cases. In addition, if there are no signs of appeal, DPC must be considered immediately when conditions such as distal limb pain, pallor, pulselessness, paraesthesia and paralysis occur during ECMO support. During the ECMO support period, our centre monitors the activated clotting time (ACT) every 4 h, maintaining it at 160–180 s, and activated partial thromboplastin time (APTT) every 4–8 h, maintaining it at 50–80 s, even after the DPC is inserted [6]. At the same time, blood supply to the lower limbs is closely monitored, the DPC pressure drop is measured, and intravascular ultrasonography is performed every 12 h. In cases of abnormalities, a multidisciplinary team (MDT) consultation is organized to consider whether vascular repair and salvage thrombectomy should be performed after removal of the ECMO cannula.

The cause of vascular variation in this patient was thought to be a congenital vascular abnormality. While establishing vascular access, incision of the skin to separate the vessels showed that there was insufficient dissociation of the CFA below the inguinal ligament to catheterize the CFA. Then, an arterial cannula (Maquet®, 23 cm, 16 F) was placed in the SFA towards the heart, and simultaneously,the DPC was placed in the SFA towards the distal limb. In the SFA, turbulence, stasis and thrombosis can easily form between the arterial cannula and DPC. In addition, the patient had a history of diabetic foot and right foot (second toe) amputation. Even with the short duration of ECMO support, there was an adverse outcome of left toe necrosis and toe amputation [7]. We reported only complications that occurred during hospitalization and did not follow up with other intubation-related complications, such as sensory disturbances of lower limb movement, that may occur or persist long after discharge.

## Conclusions

High bifurcation of the DFA is rare in clinical practice (Especially above the inguinal ligament and in the abdominal cavity), and patients with this variation are less likely to receive V-A ECMO treatment. The most lethal risk with ECMO treatment is vascular complications. Vascular complications include adverse intubation events (vascular perforation, arterial dissection, bleeding at intubation site) and extubation complications (persistent bleeding or pseudoaneurysm, arteriovenous fistula, etc.). Although it is sometimes difficult to have sufficient time to complete the vascular assessment in advance during the extracorporeal cardiopulmonary resuscitation (E-CPR) process, we should always be aware of the presence of such vascular abnormalities. Ultrasound can guide us in puncturing and intubating the patient. This is especially important when inserting a larger diameter cannula. Percutaneous arterial catheterization should not be performed if the course and anatomical location of the vessels cannot be distinguished by ultrasound. Continuous near-infrared spectroscopy (NIRS) reduces the incidence of distal limb ischaemia and can be applied to patients at higher risk (female sex, high VIS and high Sequential Organ Failure Assessment (SOFA) score). Once this vascular variation is detected during arterial cannula or DPC catheterization, the surgeon must remain vigilant and cut through the skin to expose the blood vessels and insert the cannula, and an arterial cannula with the smallest possible diameter should be chosen. In addition, the novel bidirectional perfusion cannula introduced by Marasco et al. is worth recommending [8], as it provides bidirectional blood flow. Anticoagulation should be increased and monitored during ECMO support. Even though percutaneous catheterization is widely used, surgical repair of blood vessels is still necessary in cases of local infection, ischaemic necrosis of limbs, and vascular complications. Our ECMO centre strongly recommends that 5000 IU heparin should be injected into the perfusion cannula during the withdrawal of ECMO. Distal vascular examination and arterial repair were carried out after disconnection. After the perfusion cannula was removed, arterial haemorrhage was stopped with either an occluder or surgery. Ultrasound should be performed to rule out a pseudoaneurysm or arteriovenous fistula within a few days [9]. When necessary, a superficial femoral artery remedial dredge is performed.

## Data Availability

There are no data in this manuscript.
